# Functional neurological disorder 2.0?

**DOI:** 10.1093/braincomms/fcaa217

**Published:** 2020-12-02

**Authors:** Jon Stone

**Affiliations:** Centre for Clinical Brain Sciences, University of Edinburgh, EH16 4SB, UK

## Abstract

This scientific commentary refers to ‘Briquet syndrome revisited: implications for functional neurological disorder’, by Maggio et al. (https://doi.org/10.1093/braincomms/fcaa156)

**This scientific commentary refers to ‘Briquet syndrome revisited: implications for functional neurological disorder’, by Maggio et al. (https://doi.org/10.1093/braincomms/fcaa156)**

The person with functional neurological disorder (FND) normally has a lot more to contend with than a tremor, a weak leg or a seizure. Now that personal stories of FND appear through patient organizations, on social media and in the press, something that has long been clear to health professionals who work in this area is also clear to everyone else. FND does not like to travel alone, it usually brings pain, fatigue, poor concentration, sleep disturbance and a host of other symptoms with it. In many patients, FND symptoms are not even the worst problem they have (Věchetová *et al.*, 2018). Which is worse? Agonizing constant generalized pain or a slightly weak leg that gives way sometimes? Severe incapacitating daily fatigue, or brief FND-related seizure every couple of months?

In this issue of *Brain Comms*, Maggio *et al.* (2020) review the overlap between FND and comorbid functional somatic symptoms, especially through the previous entity of ‘somatization disorder’. The name ‘Somatization Disorder’ and some of its constructs, notably an asymmetrical emphasis on female sexual symptoms are now theoretically and ethically problematic, but it did at least give a place for a recognizable condition characterized by longstanding vulnerability to repeated and different types of functional disorder. The authors remind us of Briquet, and his remarkable 1859 study, which showed in 430 individuals, that such disorders were as clinically recognizable as they are today. One of the strengths of the FND diagnosis is that, despite its stigmatizing history, the rich historical literature shows how consistent its clinical features have been across time and remain, across the world. Despite the ‘H-word’, Briquet’s approach was more modern and multiperspective than much 20th-century work. He used a model of predisposing, precipitating and perpetuating factors which was both psychological, biological and brain-based. He knew that FND and other bodily symptoms like pain were partners. So did those that devised the somatization disorder category, and so do modern patients and researchers.

The Diagnostic and Statistical Manual of Mental Disorders, 5th edition (DSM-5), currently performs poorly in capturing this complexity. In the transition from DSM-IV an awkward, and paradoxical evolution occurred. For FND, there was a new emphasis on positive diagnosis, with ‘rule in’ features, and abandonment of any psychological diagnostic criteria—because the latter does not apply to everyone. For all other bodily symptoms, as part of Somatic Symptom Disorder, there was abandonment of any need to assess whether symptoms were part of a functional disorder, and instead, a requirement to judge whether someone’s cognitions, anxiety and behaviour are disproportionate to the ‘seriousness’ of their symptoms. I personally struggle with Somatic Symptom Disorder for many reasons. Chronic pain and fatigue *are* usually serious, and *especially* when due to a functional disorder. For many patients, ‘disproportionate’ behaviour is generated by healthcare systems and societal attitudes that are badly equipped to diagnose, explain and treat their conditions. Judging disproportionality, with the exception of severe health anxiety—which the patient normally recognizes as a problem, carries a high risk of erroneous value judgement about what is a ‘serious’ condition, based on the presence or absence of identifiable structural pathology. Both FND and Somatic Symptom Disorder are trying to make sense of common disabling problems using positive diagnostic criteria, but they do so in such radically different ways that they arguably should not be on the same diagnostic axis of DSM.

The change in DSM-5 has also led to a separate problem. How should we classify conditions described above where pain, fatigue and FND vie for dominance in the clinical picture and yet it is clear, from the way they worsen and improve together, that they are facets of the same overall disorder? If the patient’s pain and fatigue do not meet the criteria for Somatic Symptom Disorder then should they be recorded on Axis-3 of DSM-5 as medical comorbidities or using a different system such as the new International Classification of Diseases, 11th revision (ICD-11), Chronic Pain codes under MG30?

Such taxonomical anxiety may seem obscure, but classification matters. It matters to patients and their families who want to know what the name of their disorder is and where their symptoms are coming from. And it matters for research and training if we want to work out how to understand and treat these disorders better in the future.

Maggio *et al.* (2020) propose squaring this difficult circle with a suggestion to modify the existing DSM-5 FND category with specifiers for pain, fatigue and mixed somatic symptoms. For the FND research community, this could help researchers be more confident that they are studying similar patients. For patients, it would acknowledge that their motor and sensory symptoms are only part of the problem. And in some health care systems, reliant on coding for reimbursement of services, it may help patients access treatment for all their symptoms, without having to be squeezed in multiple diagnostic categories. As with any classificatory system, there are problems which the authors acknowledge. What if someone mostly has pain and minor FND? Should there be a counterpart Pain diagnosis with an FND specifier? Should clinicians attempt to judge whether other somatic symptoms relate to a functional disorder or another cause such as rheumatoid arthritis. Fibromyalgia, functional gastrointestinal disorders and functional bladder disorders all have their own diagnostic criteria that do allow such diagnoses to be made. The authors suggest secondary specifiers to resolve this issue. I am personally not convinced that it is feasible, or perhaps even desirable to operationalize ‘symptom-related cognitive behavioural (psychological) features’ as a *diagnostic* subcategory, rather than as part of an assessment formulation. For me, this leads back to the same issues as Somatic Symptom Disorder. Surely everyone has symptom-related psychological features to some extent, whether they have flu or cancer? Another issue could be how common these specifiers are. The majority of my current FND patients would have a ‘mixed somatic symptoms’ category (Stone *et al.*, 2010), although through a much smaller percentage would have had Briquet’s longer duration syndrome. Stricter descriptions could help improve that.

I congratulate the authors for taking on this problem and welcome this debate, which is often avoided for territorial and other reasons, often to the detriment of patients. A good example of that is the very clear clinical overlap between FND and Complex Regional Pain Syndrome, which for years has been the subject of warfare between groups of clinicians with often polarized psychological versus biological views when reconciliation along non-dualist neuroscience based lines is now within reach (Popkirov *et al.*, 2019).

My own view is that a modification such as this could be a useful temporary sticking plaster, but it would not disentangle the deeper problems with the Somatic Disorders category of DSM-5. Other functional disorders have solved this by ‘declaring independence' from DSM. In the case of Pain Medicine this happened decades ago, for functional gastrointestinal disorders, the first Rome Foundation criteria (now adopted wholesale by ICD-11) were created in 1989, and the Bárány society developed diagnostic criteria for Persistent Postural Perceptual Dizziness in 2017. Yet none of these individual disciplines have taken on, from a classificatory perspective, the challenge of frequent functional disorder comorbidity, their shared epidemiology and mechanisms.

A recent proposal from the EURONET-SOMA group suggests a system in which functional disorders occupy their own distinct space separate from mental disorders and somatic diseases (Burton *et al.*, 2020). In this arrangement, a functional disorder could be described as single symptom (e.g. functional leg weakness), a single disorder (e.g. irritable bowel syndrome) or as a ‘multisystem functional disorder’. That feels like a good match clinically and would support a strong research framework that highlights different functional disorders but also allows them to be brought together when it makes sense to do so. A third ‘supercategory’ of functional disorders introduces, however, some problematic additional splitting when we should arguably be trying to integrate these disorders into the mainstream and recognizing their status as medical conditions with their own pathophysiology.

If you follow a logical process to try to solve these issues, then we should not have separate ‘mental’ and ‘disease’ classifications at all, and certainly not separate psychiatric and neurological ones that still persist in ICD-11—the division of which causes problems across a whole range of conditions from dementia to schizophrenia (Perez *et al.*, 2018). Ideally, functional disorders, mental disorders and those based on the more overtly structural disease would share the same space and status as medical conditions. That may seem utopian, but perhaps something to work towards—as this proposal does, one step at a time.

## Competing interests

J.S. was an advisor specifically in relation to FND for DSM-5 and has revised this specific section for an upcoming DSM-5 text revision. He is co-chair of a new Functional Neurological Disorder Society (FNDS) Classification Committee (fndsociety.org).

## Funding

J.S. is supported by a National Research Scotland Career Fellowship.

## Data availability

Data sharing is not applicable to this article as no new data were created or analysed.

## Notes

This scientific commentary refers to ‘Briquet syndrome revisited: implications for functional neurological disorder’, by Maggio *et al.* (https://doi.org/10.1093/braincomms/fcaa156).
